# Hybrid Convolutional‐Gated Recurrent Neural Network for Robust Mobile Health Activities Classification

**DOI:** 10.1155/ijta/1465162

**Published:** 2026-07-27

**Authors:** Raed Alotaibi, Omar Reyad, Mohamed Esmail Karar

**Affiliations:** ^1^ Applied College, Shaqra University, Shaqra, Saudi Arabia, su.edu.sa; ^2^ Department of Information Systems, College of Computing and Information Technology, Shaqra University, Shaqra, Saudi Arabia, su.edu.sa; ^3^ Department of Computer Science, Faculty of Computers and Artificial Intelligence, Sohag University, Sohag, Egypt, sohag-univ.edu.eg; ^4^ Department of Industrial Electronics and Control Engineering, Faculty of Electronic Engineering, Menoufia University, Menouf, Egypt, menofia.edu.eg

**Keywords:** artificial intelligence, body motion signals, convolutional neural network, gated-recurrent unit, mobile health

## Abstract

Mobile health has become a popular option for patients to monitor and analyze their body activities and vital signs using their mobile devices, such as smartphones and smartwatches. In addition, the healthcare community has begun using artificial intelligence (AI) models to automate the diagnosis of abnormal conditions and diseases, particularly in real‐time emergency scenarios. This article introduces a new deep learning model to automatically identify daily body activities. We propose a hybrid AI model that combines a convolutional neural network (CNN) and a gated recurrent unit (GRU) for robust human activity classification. Key contributions include (1) the design of an end‐to‐end CNN‐GRU model that jointly extracts local spatiotemporal features and models long‐term temporal dependencies from raw sensor data, and (2) a comprehensive benchmarking framework validating the developed model against previous machine learning and deep learning classifiers. A public mobile health dataset (MHEALTH) has been used in this study. This dataset includes 12 physical activities, for example, knee bending, walking, and running. Key findings demonstrate that the CNN‐GRU model achieves a state‐of‐the‐art classification accuracy of 99.50% on the publicly available MHEALTH dataset, which encompasses 12 distinct physical activities. It significantly outperforms CNN‐LSTM (98.83%), 1‐D CNN (96.89%), and traditional ensemble methods while maintaining high precision, recall, and F1‐scores across all activity classes. Therefore, our developed model can be implemented in a cloud computing system to monitor senior patients as a critical healthcare application.

## 1. Introduction

Mobile health (m‐health) leverages mobile gadgets such as smartphones, tablets, and wearable technologies to monitor and improve health outcomes. With the increasing ubiquity of mobile devices, m‐health has emerged as a powerful tool in modern healthcare, offering accessibility, real‐time monitoring, and personalized health management [1]. The concept of m‐health was introduced in 2004 by the authors in [2]. Using mobile and wireless technologies, m‐health provides health services anywhere, anytime, and to any person without constraints in location and time [3]. Also, m‐health refers to the use of telemedicine and other technologies to provide healthcare services to patients online [4]. It has been claimed that m‐health remains in an evolutionary phase, with ongoing integration of AI, IoT, and edge computing required to achieve scalable, interoperable, and clinically validated deployments [1, 5, 6]. M‐health offers several advantages, such as helping patients manage their health independently and minimizing the cost of providing health services, especially in villages and rural areas [5]. Additionally, m‐health can deliver emergency health services quickly, extend health services, manage and prevent chronic conditions, and assist with diagnostic procedures. Nowadays, with technological advances, m‐health enables doctors to access medical information easily, anywhere, anytime, and supports them in checking patients′ health status remotely [6].

**Figure 1:**
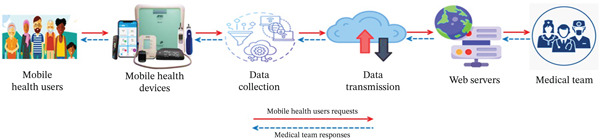
A standard mobile health architecture including the data flow from wearable sensors to cloud‐based processing to assist the remote healthcare by medical staff.

There are some challenges of m‐health in developing countries, such as the rarity of skilled medical staff, insufficient infrastructure, cost barriers, limitations for crowdsourcing, and phone battery life. Therefore, it has been recommended that educational activities in m‐health can help medical practitioners utilize mobile technologies to provide healthcare [7]. One of the activities supported by m‐health technologies is promoting health policies to prevent cardiovascular diseases (CVD) through amateur sports activities (PASA) [8]. A depiction of a standard m‐health architecture is given in Figure 1, in which data from various user mobile devices is sent through the network to remote servers for healthcare medical teams to process and respond to all users′ requests.

In human activity recognition (HAR) and the application of recognition models, ML‐based techniques have become powerful instruments for prediction and classification [9]. To accomplish HAR goals, researchers have widely used traditional ML methods, such as random forest (RF) [10] and support vector machine (SVM) [11]. Conventional activity detection techniques rely on manually extracted features from sensor data, often using ML algorithms that rely on statistical and/or structural characteristics such as medians, mean values, and standard deviations. Subject‐matter expertise is frequently required to extract the most pertinent manual elements. Although these custom features perform adequately in situations with little training data, the extraction process becomes more complex as the number of sensors increases [12]. Moreover, deep learning (DL) has become quite popular because of its powerful capabilities to extract features and conduct classification simultaneously. DL processes data and solves complex problems using multilayered artificial neural networks, which differ from typical ML techniques [13]. A variety of DL models, such as recurrent neural networks (RNNs), CNNs, long short‐term memory (LSTM), and gated recurrent units (GRUs), have demonstrated promising outcomes across a wide range of applications. CNN uses weight‐sharing and convolving filters to overcome high dimensionality effectively [13]. However, RNNs are a type of neural network particularly effective for managing time series data and sequential tasks, as they leverage past outcomes as inputs for the current step and incorporate hidden states [14]. GRUs address the vanishing‐gradient issues that can occur in standard RNNs, where gradients become very small during backpropagation, making it difficult for the model to learn long‐term dependencies in the data [15, 16]. Compared with classic ML techniques, DL techniques offer clear benefits by efficiently handling massive datasets and overcoming the drawbacks of manual feature extraction. Furthermore, the CNN‐GRU model identifies local patterns—such as short movement bursts and sensor readings—through its convolutional layers and detects long‐term trends—such as changes in activity over time—through its GRU layers. This combination enables the model to identify complex physical activities more accurately and with greater context.

Mobile devices equipped with DL algorithms via cloud‐based services can enable real‐time diagnostics, such as detecting early signs of diseases such as pancreatic cancer [17] and diabetic retinopathy [18]. The role of m‐health, based on DL, looks promising, with ongoing advancements in model efficiency and integration with other AI technologies in digital health [19]. As mobile devices become more powerful and DL techniques continue to advance, one can expect even more sophisticated and accessible healthcare solutions that leverage DL′s unique capabilities in m‐health. These developments will lead to more proactive, personalized, and preventative healthcare on a global scale.

Despite promising advancements in m‐health and DL‐based HAR, some critical challenges remain as follows: First, wearable sensor data exhibit significant heterogeneity due to variations in sensor placement, sampling rates, and individual user characteristics, which often limits the generalizability of models across diverse populations. Second, achieving real‐time activity recognition on resource‐constrained mobile or edge devices requires lightweight architectures that maintain high accuracy while minimizing computational overhead and power consumption. Third, daily activities share highly similar motion features (e.g., knee bending vs. climbing stairs), creating temporal ambiguity that challenges robust discrimination. Finally, continuous sensor data collection and cloud‐dependent processing raise ongoing concerns regarding data privacy, security, and inference latency, necessitating efficient on‐device deployment strategies. Addressing these challenges motivates the design of our proposed lightweight classifier.

Thus, this work introduces a novel DL model designed for the automatic recognition of patients′ physical activities. The study′s primary contributions are as follows:

• Developing a novel CNN‐GRU model to automatically classify 12 physical human body activities from the precollected MHEALTH dataset using multimodal sensing, and
• Performing a comparative analysis to validate the competitive performance of our new hybrid model against other ML and DL classifiers from previous research.

The rest of this manuscript is structured as follows: Section 2 presents a review of the relevant methods for HAR; the third section details the technique employed in our developed CNN‐GRU model; the fourth section details the experiments and the evaluation of this study; and the fifth and sixth sections give the discussion and conclusion of this paper′s findings along with the future work, respectively.

## 2. Related Work

Identifying m‐health activities using DL is a rapidly growing area of research. These activities typically include recognizing physical movements, monitoring health behaviors, and detecting patterns in user interactions with mobile devices or wearables. Here is a summary of related works in this domain, highlighting significant contributions and methodologies. HAR is a core application of DL in m‐health. The goal is to automatically identify activities like walking, running, sitting, or sleeping using data from sensors embedded in smartphones and wearables. The method proposed in [20] begins by extracting features using the S transform (ST), followed by learning a low‐dimensional substantial trait impersonation from the authentic feature subspace through the supervised regularization‐based robust subspace (SRRS) technique. SRRS enhances the learning process by reducing noise and redundancy, allowing for extra solid and discriminative features that improve sample descriptions. To extract and preserve various latent patterns of activities, the work in [21] provided a semisupervised framework that is balanced in terms of patterns. It takes advantage of the independence of several sensory modalities. It uses recurrent convolutional attention networks (RCANs) to carefully select salient regions from the input that are indicative of human behavior. With 10% labeled training data, their proposed model achieves competitive performance against numerous semisupervised and supervised models. The study in [22] suggested a deep RNN method based on sequential input in a body sensor‐based system in order to identify behaviors. This method combined data from many body sensors, including magnetometers, accelerometers, and electrocardiograms (ECGs). Kernel principal component analysis, in turn, is used to refine the collected features (KPCA) further. After the training of an activity RNN with robust characteristics, behavior recognition is achieved. The experimental results demonstrate that this strategy works better than other techniques currently in use, with a 99% accuracy rate. In [23], the authors used a method to capture patterns across different temporal scales in datasets by employing a deep CNN (DCNN) ensemble. This approach is particularly effective because the data naturally exists as a temporal sequence. Extracting information at multiple scales provides valuable insights into user activities, enabling the identification of basic motion patterns, such as wrist twists when picking up a spoon, as well as more complex movements, such as human gait. The work presented in [24] introduced a deep ConvLSTM structure that takes into account the spatiotemporal characteristics of raw data collected from diversified wearable sensors. It employs a self‐attention manner to detect meaningful compositions of embeddings for decoding human activities, making it suitable for HAR and also scalable for numerous instances of sensors and time periods. In [25], a hybrid DL model was proposed that integrates a LSTM network with a CNN and is enhanced with a self‐attention mechanism to improve predictive performance. This hybrid model achieved an accuracy of 98.76% on the MHEALTH dataset, as described below in Section 3, which demonstrates its effectiveness in HAR. The study in [26] suggested a secured framework, known as the edge of things, suitable for smart healthcare applications, specifically tailored for health real‐time monitoring while preserving sensitive healthcare data safeguard and confidentiality. The framework incorporates a K‐medoids–based nearest neighbors (KMNN) clustering technique for analyzing biosignal data to detect abnormalities, along with attribute‐based encryption (ABE) to secure biosignal data series and enable secure accessibility. Experimental results showed enhanced performance, achieving up to 98.5% accuracy while maintaining data security. To extract both spatial and temporal features from raw sensor data, the DL‐based HAR model presented in [27] integrates CNN layers with bidirectional long short‐term memory (Bi‐LSTM) units. The Rao‐3 algorithm for optimization was utilized to determine the optimal hyperparameters, resulting in an accuracy of 99.25% on the MHEALTH datasets. Finally, the authors in [28] examined the efficacy of deep neural network (DNN) models for activity detection in individuals with Parkinson′s disease (PD) using wearable sensor data. Their approach looked closely at the impacts of domain adaptation, manual axis reorientation, data augmentation, and model complexity. The results indicated that a moderately complex model, trained on the expanded PAMAP2 dataset and adjusted using the domain adversarial neural network (DANN) domain adaptation method to align with the PD domain, achieved the highest performance in activity recognition. Table 1 summarizes related work on MHEALTH dataset‐based DL research methods, highlighting their pros and cons.

**Table 1:** Related works regarding MHEALTH dataset‐based DL research methods.

Study	Method	Advantages	Disadvantages	Accuracy rate
Lu et al. [20]	SRRS	Robust low‐dimensional feature representation.	Recognition performance of user‐independent is worse than user‐dependent cross‐validation.	96.10
Chen et al. [21]	RCAN	Semisupervised deep model for imbalanced activity recognition.	Labeling accuracy decreases with training rounds.	94.05
Uddin et al. [22]	RNN	Deep RNN‐based activity recognition system utilizing fused data from wearable body sensors.	Addressing the vanishing gradient problem that arises when modeling long sequences.	99.00
Sena et al. [23]	DCNN	Identify patterns at various temporal scales within the data.	Misclassified more than 60% of the samples.	96.27
Singh et al. [24]	ConvLSTM	Utilizes a self‐attention track to capture, choose, and learn significant time periods.	Improvements are not significant in the MHEALTH dataset.	94.86
Khatun et al. [25]	CNN‐LSTM	Provides human activity recognition and analysis in clinical trials.	Extracting deeper information is challenging with basic acceleration data.	98.76
Singh and Chatterjee [26]	KMNN	Identify anomalies while ensuring the secrecy of patient data stored in the cloud.	As users/data size increases, computational time is linearly increased.	98.5
Challa et al. [27]	CNN and Bi‐LSTM	Bi‐LSTM with CNN models are employed to improve feature extraction.	Not yielding an optimal solution for all activity scenarios.	99.25
Davidashvilly et al. [28]	CNN and DANN	Investigation of scope adaptation and data augmentation methods.	Variability in the activity matching process among small‐sized datasets.	94.98

Recent advancements continue to expand the scope of HAR beyond standalone wearable analytics toward integrated IoT and smart health ecosystems. For instance, Sethi et al. [29] proposed a DL‐driven framework for remote health monitoring within IoT‐enabled smart cities, emphasizing scalable data pipelines and continuous activity tracking for chronic disease management and elderly care. Similarly, Bijrothiya and Soni [30] introduced a feature‐driven hybrid DL architecture for HAR in holographic IoT environments, demonstrating the efficacy of combining engineered spatiotemporal features with deep networks to capture complex motion dynamics. Although these studies highlight the growing convergence of HAR with next‐generation connectivity and infrastructure, they often rely on computationally intensive pipelines, cloud‐dependent processing, or manual feature engineering. In contrast, this study develops a lightweight, end‐to‐end CNN‐GRU hybrid that automatically extracts local spatial patterns and long‐term temporal dependencies directly from raw multimodal sensor streams.

## 3. Dataset and Methods

### 3.1. Dataset Characteristics

The MHEALTH dataset has been collected by the authors in [31]. As listed in Table 2, the collected dataset consists of body motion recordings made by 10 individuals with a variety of profiles as they engaged in 12 different physical activities. The MHEALTH recordings were acquired using wearable sensors and a video camera. The sensors were attached to the subject′s left ankle, right wrist, and chest, secured in place with elastic straps. In order to better capture the dynamics of the body, many sensors are employed to monitor the motion experienced by various body sections, including acceleration, rate of turn, and magnetic field direction. A sample rate of 50 Hz is used for all sensing modalities to be suitable for recording human activities. The MHEALTH dataset gives generalized daily living activities of the body, including raising the arms in front and bending the knees. In addition, it encompasses both the intensity of the actions and the speed at which they are performed, such as sitting and relaxing and cycling and running. To prevent the missing of relevant information, no preprocessing is done on the data [31].

**Table 2:** List of physical activities in the MHEALTH dataset.

Activity no.	Description	Duration (min)
A_01_	Standing still	1.0
A_02_	Sitting and relaxing	1.0
A_03_	Lying down	1.0
A_04_	Walking	1.0
A_05_	Climbing stairs	1.0
A_06_	Waist bends forward	20.0
A_07_	Frontal elevation of arms	20.0
A_08_	Knees bending (crouching)	20.0
A_09_	Cycling	1.0
A_10_	Jogging	1.0
A_11_	Running	1.0
A_12_	Jump front and back	20.0

As detailed in Table 2, the dataset exhibits a natural imbalance in recording durations: Short‐duration activities (A01–A05 and A09–A11, each lasting 1 min) contribute approximately 8% of the total samples per class, whereas long‐duration activities (A06–A08 and A12, each lasting 20 min) account for roughly 17% each. To preserve this realistic distribution during the training‐testing split, we employed stratified sampling at the subject level rather than random instance‐level splitting. Regarding class balance, no artificial oversampling or undersampling techniques were applied. The natural frequency distribution closely reflects real‐world human activity patterns, where prolonged tasks occur less frequently than brief postural transitions. We implemented a subject‐wise partitioning strategy to prevent data leakage in time‐series and wearable sensor classification. The dataset was split into 80% for training and 20% for testing based on individual subjects rather than individual sensor windows. This ensures that all recordings from a given participant appear exclusively in either the training or testing set, eliminating cross‐subject information contamination. Furthermore, temporal ordering was preserved within each recording session; no shuffling was applied across sequential time windows to maintain the integrity of activity transitions. Finally, all feature‐scaling parameters were computed exclusively using training‐set statistics and subsequently applied to the validation and test sets, thereby preventing inadvertent information leakage from unseen data, in line with best practices for robust wearable HAR evaluation [31].

### 3.2. One‐Dimensional (1‐D) CNN

**Figure 2:**
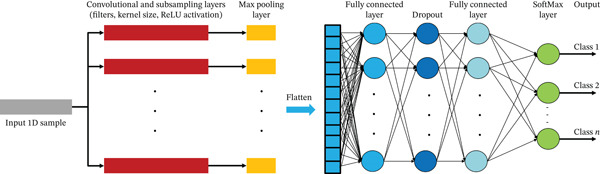
Generic structure of CNN layers for classifying multi‐*n* *-*classes.

In medical applications, CNNs are a useful tool for feature extraction and classification tasks of normal and abnormal cases [32]. Here, a 1‐D CNN has been developed as a multiclass classifier for human body activities. Generally, the structure of CNN consists of cascaded layers, including convolutional operations, regularization, subsampling, dropout, and SoftMax layers [33]. This generic structure is illustrated in Figure 2. The layer of convolutional as well as subsampling performs feature recognition on the 1‐D input sample through filtering processes utilizing kernels, convolutions, and the rectifier linear unit (ReLU). To extract the most significant features inside the entire feature mapping covered by the preset filter, the max pooling layer aggregates the data. As shown in Figure 2, the flatten layer′s purpose is to convert the multidimensional feature mapping matrix into a singular 1‐D array. Dropout serves as a regularization operation for self‐modifying the CNN structure, helping to prevent overfitting in the neural network. To produce the final result of projected classes, the SoftMax function is applied to the outputs from the fully linked network layer.

### 3.3. Basic GRU

**Figure 3:**
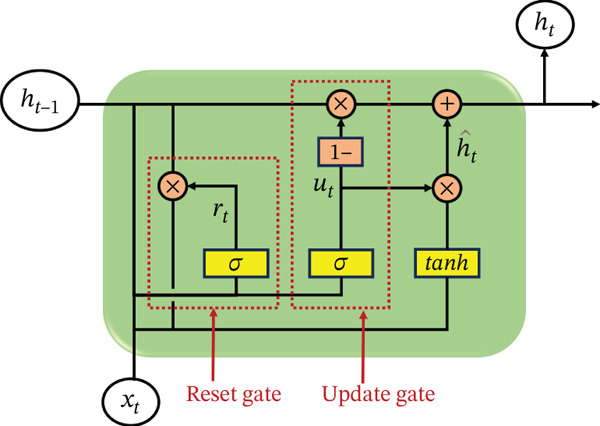
Basic gated recurrent unit (GRU) architecture.

Cho et al. [34] proposed the GRU network, which is essentially a simplified LSTM network. It is classified as a RNN to process sequential data successfully. Similar to the LSTM network, the GRU is designed to address the vanishing gradient problem without requiring a separate memory cell. Figure 3 illustrates the basic architecture of the GRU, which includes two gating layers: the reset gate and the update gate [15, 35]. The update gate specifies how much data from the previous memory can be carried forward, whereas the reset gate decides how much data from the previous memory should be destroyed. To mathematically represent the GRU, some parameters are defined, where (*t* − 1) denotes the previous timestep for the hidden state *h*_*t*−1_ and the corresponding current input *x*_*t*_. The prediction of the GRU, *h*_*t*_, is then defined as follows:

(1) ht=fxt,ht−1,

(2) Zt=σWz.ht−1,Xt+bz,

(3) rt=σWr.ht−1,Xt+br,

(4) ht~=tanhWh.rt⊙ht−1,xt+bh,

(5) ht=1−zt⊙ht−1+zt⊙ht~,

where *b*_*z*_, *b*_*r*_, and *b*_*h*_ are the bias vectors. *W*_*z*_, *W*_*r*_, and *W*_*h*_ are the weight matrices. The sigmoid *σ* and tanh functions are the activation functions, as shown in Figure 3.

### 3.4. Developed CNN‐GRU Model

**Figure 4:**
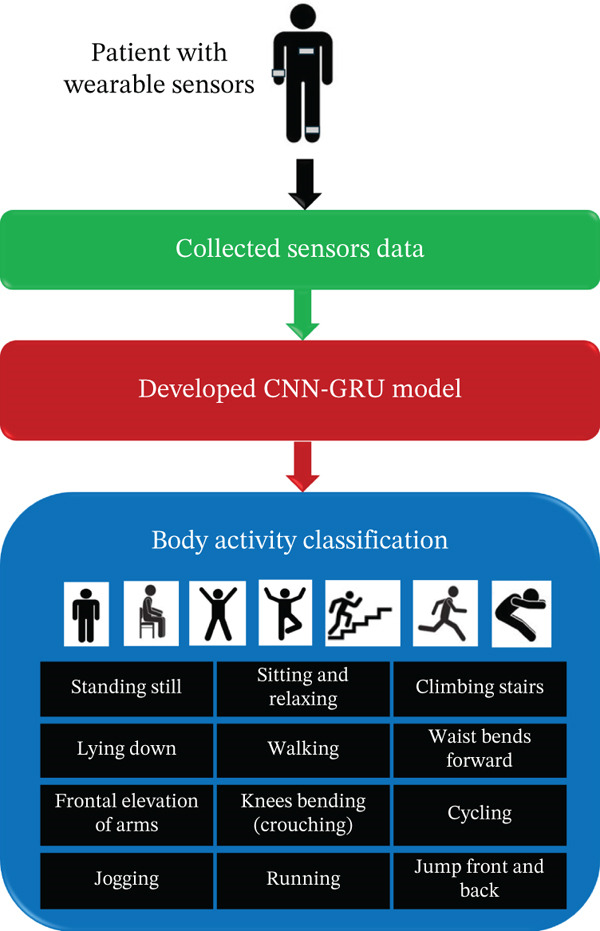
Proposed classification workflow of 12 physical body activities using the developed CNN‐GRU model.

Figure 4 shows the proposed workflow for identifying different human activities using our developed CNN‐GRU model. The wearable sensors are placed on the patient to monitor their body motion in *x*‐*y*‐*z* coordinates. Then, the recordings of the sensor data can be collected using a mobile application and/or a cloud server [12, 31]. These motion data are then input into the developed CNN‐GRU classifier to identify multiclass human activities, as given in Table 2.

Table 3 provides a detailed overview of the structural layers of the developed CNN‐GRU model. It includes two convolutional layers, two batch normalization layers, two ReLU activation functions, one max pooling layer, one GRU layer, one fully connected dense layer, and a final output SoftMax layer. Furthermore, the total number of trainable parameters in the CNN‐GRU model is 114,717. Therefore, it can be considered a lightweight deep network classifier.

**Table 3:** The layer configuration of the developed CNN‐GRU model for classifying physical human activities.

	Layer (type)	Output shape	Parameter #
	Conv1D	(100, 64)	2368
	Batch normalization	(100, 64)	256
	ReLU	(100, 64)	0
	Conv1D	(100, 128)	24,704
	Batch normalization	(100, 128)	512
	ReLU	(100, 128)	0
	Maximum pooling	(50, 128)	0
	GRU (GRU)	(128)	99,072
	Dropout (dropout = 0.5)	(128)	0
	Fully connected dense (dense)	(128)	16,512
Output layer	SoftMax	(13)	1677
Total parameters	145, 101		
Trainable parameters	144, 717		
Nontrainable parameters	384		

### 3.5. Performance Evaluation Metrics

The following evaluation metrics for classifying human health activities have been used to assess the developed CNN‐GRU model. A confusion matrix was created using cross‐validation calculations [36]. The four main predicted results of 12 physical activities, as given in Table 2, are true positive (TP), true negative (TN), false positive (FP), and false negative (FN). Consequently, accuracy, precision, recall, and F1‐score are estimated as follows:

(6) Accuracy=TP+TNTP+FP+FN+TN%.

(7) Precision=TPTP+FP.

(8) Recall=TPTP+FN.

(9) F1−score=211/recall+/precision.

## 4. Experiments

Experiments have been carried out to identify the performance of our developed CNN‐GRU model and other DCNN models in recognizing human activities using the MHEALTH dataset. Packages from TensorFlow‐Keras, web‐based interactive (Jupyter V6.4) computing notebook, and Python′s Anaconda Navigator V2.3 were used to implement all evaluated deep classifiers [37]. A laptop with an Intel Core i7 CPU, 16 GB of RAM, and an 8 GB NVIDIA GeForce GPU was used for these experiments.

**Figure 5:**
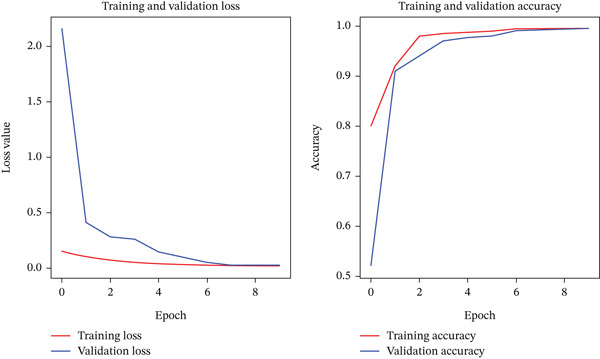
Developed CNN‐GRU model training and validation loss and accuracy versus epochs.

The human activities dataset, as shown in Table 2, was split into 80%–20% to start the training state of all tested deep classifiers. This means that the testing phase used 20% of these human activities to achieve a multiclass identification procedure of various human activity cases. The hyperparameter of the CNN‐GRU suggested model was carefully selected, as given in Table 4. For a small number of 10 epochs, Figure 5 shows successful training of the deep classifier with high training and validation accuracies of 99.92% with the minimum loss reaching zero approximately.

**Table 4:** Hyperparameter settings for the developed CNN‐GRU model.

Hyperparameter	Value
Number of epochs	10
Batch_size	155
Optimizer	Adam
Loss_function	Sparse categorical cross‐entropy
Metric	Spars categorical accuracy

**Figure 6:**
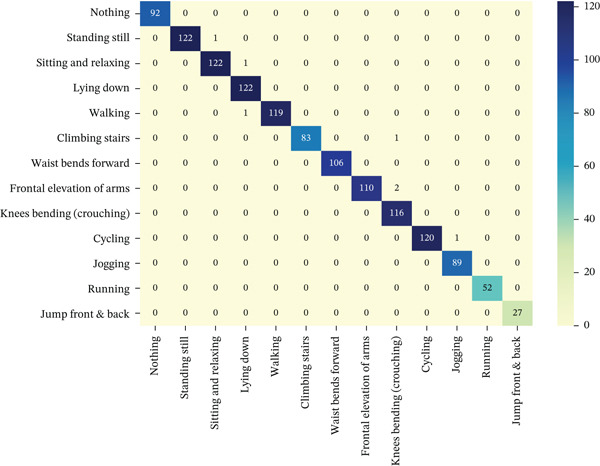
Confusion matrix of the resulting physical activities classification of the human body.

Figure 6 depicts the confusion matrix to assess the classification accomplishment of the developed CNN‐GRU model for human activities. High accuracy has been achieved in identifying human activities. Only seven samples were misclassified for A_2_—sitting and relaxing (one sample), A_3_—lying down (two samples), A_8_—knees bending or crouching (three samples), and A_10_—jogging (one sample). As given (1–4), four quantitative metrics, named precision, recall, F1‐score, and accuracy, have been employed to validate the fulfillment of all evaluated classifiers. Then, the base models, which are SVM and RF, other CNN models, namely 1‐D CNN without any additional units and CNN‐LSTM, have been performed and compared with the accomplishment of our CNN‐GRU classifier, as illustrated in Table 5. The 1‐D CNN classifier attained the lowest accuracy score of 96.89% without adding LSTM or GRU layers. The CNN‐LSTM classifier showed a good accuracy score of 98.83% and achieved the highest metric values for the A_7_ activity of frontal elevation of arms compared with the other classifiers. Nevertheless, the developed CNN‐GRU attained the best accuracy score of 99.50% and also the highest metric values for most human activities, for example, climbing stairs (A_5_) and waist bends forward (A_6_), as presented in Table 5.

**Table 5:** Comparative evaluation metrics of all tested deep classifiers for human body activities in this study.

Classifier	Metric	Human activity class												
		A_0_	A_1_	A_2_	A_3_	A_4_	A_5_	A_6_	A_7_	A_8_	A_9_	A_10_	A_11_	A_12_
SVM	Precision	0.92	0.94	0.89	0.88	0.96	0.93	0.97	0.95	0.92	0.85	0.86	0.82	0.93
	Recall	0.92	0.95	0.87	0.90	0.95	0.94	0.98	0.93	0.91	0.83	0.84	0.80	0.94
	F1‐score	0.92	0.95	0.88	0.89	0.96	0.94	0.97	0.94	0.91	0.84	0.85	0.81	0.94
	Accuracy	92.28												
Random forest	Precision	0.96	0.92	0.91	0.97	0.96	0.99	0.95	0.93	0.89	0.90	0.87	0.96	0.94
	Recall	0.97	0.91	0.93	0.98	0.96	0.99	0.94	0.93	0.87	0.88	0.85	0.97	0.92
	F1‐score	0.97	0.92	0.92	0.97	0.96	0.99	0.95	0.93	0.88	0.89	0.86	0.97	0.93
	Accuracy	94.79												
CNN	Precision	**1.0**	0.99	**0.99**	0.98	0.98	0.89	0.91	0.99	**0.98**	0.98	0.93	0.96	**1.0**
	Recall	**1.0**	0.98	0.98	**1.0**	0.97	0.98	0.97	0.98	0.84	**0.99**	**1.0**	0.92	0.89
	F1‐score	**1.0**	0.99	**0.99**	0.99	0.98	0.93	0.94	0.99	0.91	0.99	0.96	0.94	0.94
	Accuracy	96.89												
CNN‐LSTM	Precision	**1.0**	0.99	0.98	**1.0**	0.99	0.99	0.95	**1.0**	0.97	**1.0**	0.98	**1.0**	**1.0**
	Recall	**1.0**	**0.99**	**1.0**	0.99	**1.0**	0.96	0.97	**1.0**	0.96	**0.99**	**1.0**	0.98	**1.0**
	F1‐score	**1.0**	0.99	0.99	**1.0**	**1.0**	0.98	0.96	**1.0**	0.97	**1.0**	**0.99**	0.99	**1.0**
	Accuracy	98.83												
Developed CNN‐GRU	Precision	**1.0**	**1.0**	**0.99**	0.98	**1.0**	**1.0**	**1.0**	**1.0**	0.97	**1.0**	**0.99**	**1.0**	**1.0**
	Recall	**1.0**	**0.99**	0.99	**1.0**	0.99	**0.99**	**1.0**	0.98	**1.0**	**0.99**	**1.0**	**1.0**	**1.0**
	F1‐score	**1.0**	**1.0**	**0.99**	0.99	**1.0**	**0.99**	**1.0**	0.99	**0.99**	**1.0**	**0.99**	**1.0**	**1.0**
	Accuracy	**99.50** ^∗^												

^∗^Best performance values are indicated in bold. Symbols of human activity classes are predefined in the context.

Using the MHEALTH dataset, Table 6 illustrates a comparative evaluation of our developed CNN‐GRU with the most well‐known classifiers for automated identification of human activities. The multilayer perceptron (MLP) classifier presented the worst case of a high accuracy score of less than 90% [38]. To improve the classification performance of ML classifiers, Catal et al. [39] proposed an ensemble model of MLP, decision tree, and logistic regression classifiers. It achieved a slightly higher accuracy score of 91.72 than the MLP. A combined CNN‐LSTM classifier has been proposed in [40], achieving 93.80% accuracy. Our developed CNN‐GRU is still superior to these previous classifiers and achieved a better accuracy score of 99.50%, as listed in Table 6.

**Table 6:** Comparison between the developed CNN‐GRU and previous classifiers using the same dataset in this study.

Study	Classifier	Accuracy (%)	Precision	Recall	F1‐score	AUC
Kwapisz et al. [38]	MLP	89.49	0.88	0.87	0.87	—
Catal et al. [39]	Ensemble	91.72	0.90	0.91	0.90	—
Ordóñez and Roggen [40]	CNN‐LSTM	93.80	0.92	0.93	0.92	0.985
Developed CNN‐GRU		**99.50** ∗	**0.994**	**0.993**	**0.993**	**0.998**

^∗^Best performance values indicated in bold.

### 4.1. Ablation Study

To validate the architectural design choices and quantify each component′s contribution to the model′s overall performance, we conducted a systematic ablation study. Five structural variants were evaluated under identical experimental conditions (80/20 subject‐wise split, Adam optimizer, batch size 155, 10 epochs, sparse categorical cross‐entropy loss). The variants isolate the impact of temporal modeling units, regularization mechanisms, and parameter efficiency. Table 7 summarizes the comparative results across key metrics.

**Table 7:** Ablation study results comparing architectural model variants on the MHEALTH dataset.

Model variants	Dropout rate	Accuracy (%)	F1‐score	Trainable parameters (K)	Inference time (ms/sample)
CNN	0.0	96.89	0.962	27.3	8.1
CNN‐LSTM	0.0	98.83	0.984	152.4	15.7
CNN‐GRU	0.0	98.91	0.985	144.7	12.1
CNN‐GRU	0.3	99.21	0.990	144.7	12.2
Developed CNN‐GRU	0.5	99.50	0.993	144.7	12.3

CNN only achieved 96.89% accuracy, confirming that spatial feature extraction alone is insufficient to distinguish temporally complex activities (e.g., knee bending vs. climbing stairs). Introducing a recurrent unit (CNN‐LSTM and CNN‐GRUs) consistently improved accuracy by > 2%, demonstrating that modeling sequential dependencies is essential for robust HAR. CNN‐GRU without dropout outperformed CNN‐LSTM by 0.08% while requiring 5% fewer trainable parameters and 22% faster inference. This aligns with the theoretical advantage of GRU′s simplified two‐gate mechanism, which reduces computational overhead without sacrificing the capacity to capture midrange temporal dependencies typical of wearable sensor windows. Comparing variants of CNN‐GRUs reveals that dropout is critical for generalization. Omitting dropout in CNN‐GRU yields high training accuracy, but validation performance plateaus due to mild overfitting on short‐duration activities. A dropout rate of 0.3 for the CNN‐GRU model improves stability, but a dropout rate of 0.5 achieves the highest test accuracy (99.50%) and F1‐score (0.993) by optimally balancing representational capacity and regularization. Crucially, this performance gain is achieved without increasing model size or inference latency. This ablation study validates that the developed CNN‐GRU architecture with a 0.5 dropout rate represents the optimal trade‐off between classification accuracy, computational efficiency, and generalization capability. These findings justify the final design selection and reinforce the model′s suitability for deployment in resource‐constrained m‐health environments.

## 5. Discussion

Utilization of AI‐based models for enhancing healthcare services, such as human activity identification, has recently gained more attention due to the powerful capabilities of precision diagnostics and automating repetitive medical tasks, thereby saving time for patients and medical staff. Here, the results of our developed CNN‐GRU demonstrate successful performance in classifying 12 physical human activities, as shown in Figure 6. Moreover, Tables 5 and 6 illustrate the outperformance of the developed CNN‐GRU over MLP, 1‐D CNN, and CNN‐LSTM classifiers reported in previous studies, achieving an accuracy of 99.50%. As further validated by the ablation study in Table 7, the GRU layer in the developed model (see Table 3) significantly enhances the classification accuracy score of 1‐D CNN from 96.89% to 99.50%, as presented in Table 5. Although the CNN‐LSTM classifier showed a similar performance to the developed CNN‐GRU with an accuracy score of 98.83%, the GRU layer achieved better performance than the LSTM because of its simple structure and faster training speed on the MHEALTH dataset, as illustrated in Table 5. Therefore, the CNN‐GRU classifier is efficient to run on mobile devices with minimal computing resources and serves as an accurate, lightweight DL model for human activity identification.

A critical consideration for real‐world m‐health deployment is model efficiency. The proposed CNN‐GRU architecture comprises only 144,717 trainable parameters, positioning it as a lightweight alternative to large‐scale pretrained DL models. Although foundation and pretrained networks (e.g., Vision Transformers, ResNet variants, or large time‐series encoders) often contain millions to billions of parameters, they are predominantly optimized for 2‐D imagery or natural language processing, leading to domain mismatch and negative transfer when applied to 1‐D multimodal wearable signals. Furthermore, such heavy models demand substantial memory, computational power, and continuous cloud connectivity, which contradicts the latency, privacy, and battery constraints of real‐world m‐health systems. By contrast, our purpose‐built CNN‐GRU is trained from scratch on domain‐specific sensor dynamics, eliminating the need for costly architectural adaptation or fine‐tuning while maintaining a minimal footprint. The model achieves a state‐of‐the‐art accuracy of 99.50% with < 150 K parameters, demonstrating that a streamlined, task‐optimized architecture can surpass overparameterized pretrained alternatives in both classification performance and edge‐deployment feasibility for continuous remote patient monitoring. The number and placement of wearable sensors on the body are crucial for data acquisition and human activity identification. Also, the performance of the CNN‐GRU model is constrained by data quality, model complexity, generalization ability, and the inherent similarity among certain activities, such as knee bending and climbing stairs. Hence, there are many relevant human activity datasets such as the University of Southern California Human Activity Dataset (USC‐HAD) [41] and UNIMIB‐SHAR dataset [42]. Here, the MHEALTH dataset [31] has been used to test and validate the developed CNN‐GRU, but it is flexible and can be applied to other HAR datasets after adjusting the number of targeted human activity classes and retraining the classifier. Additionally, the hyperparameter values of the developed CNN‐GRU can be optimized and automatically selected by incorporating a metaheuristic optimization algorithm, for example, hierarchical particle swarm optimization (H‐PSO) [43] with an additional explanation module [44]. However, our developed CNN‐GRU model is still valid to accurately automate the identification of human activities, as illustrated in Table 6.

## 6. Conclusions and Outlook

This article introduced a new and effective CNN‐GRU model for multiclass classification of human activities utilizing wearable sensors. The classification results group the human activities into 12 distinct physical body activities, as depicted in Figure 4. The developed CNN‐GRU showed superior performance over 1‐D CNN and CNN‐LSTM classifiers, as illustrated in Table 5. Moreover, it achieved the best accuracy score of 99.50% compared with the state‐of‐the‐art ML and CNN‐LSTM classifiers, as presented in the Table 6.

The ultimate goal of this research work is to develop a robust m‐health application for real‐world deployment in digital health systems. Our future work will involve integrating the hybrid CNN‐GRU model into edge‐enabled wearable platforms, such as commercial smartwatches, to enable real‐time recognition of physical activities. By leveraging the ultrareliable, high‐bandwidth capabilities of emerging 5G and 6G communication networks, coupled with scalable cloud and edge computing infrastructures, the proposed m‐health system can support continuous remote monitoring and large‐scale online clinical trials. This integration holds transformative potential for personalized healthcare, chronic neurodegenerative disease management, such as PD, rehabilitation tracking, and elderly care, empowering clinicians and medical staff with actionable, data‐driven insights while enhancing patient quality of life. Therefore, we will focus on model compression for on‐device inference, cross‐dataset generalization, and federated learning strategies to preserve user privacy in distributed healthcare IoT systems.

## Author Contributions

M.E.K.: conceptualization, idea proposal, methodology, results, writing, submission, and preparation; R.A.: writing, data curation, editing, and supervision; and O.R.: writing, editing, review, and visualization. All authors have read and agreed to this version of the manuscript.

## Funding

No funding was received for this manuscript.

## Conflicts of Interest

The authors declare no conflicts of interest.

## Data Availability

The data that support the findings of this study are openly available in UC Irvine Machine Learning Repository at 10.24432/C5TW22 (Reference Number 10.24432/C5TW22).
